# Kisspeptin-13 Improves Spatial Memory Consolidation and Retrieval against Amyloid-β Pathology

**DOI:** 10.22037/ijpr.2019.112199.13599

**Published:** 2019

**Authors:** Shima Ebrahimi Khonacha, Mahyar Janahmadi, Fereshteh Motamedi

**Affiliations:** a *Neuroscience Research Center, School of Medicine, Shahid Beheshti University of Medical Sciences, Tehran, Iran. *; b *Department of Physiology, School of Medicine, Shahid Beheshti University of Medical Sciences, Tehran, Iran.*

**Keywords:** Kisspeptin, Alzheimer, Spatial Memory, Morris water maze, Rat

## Abstract

It has been shown that brain glucose metabolism impairment, obesity, and diabetes could lead to cognitive decline and Alzheimer’s disease (AD) pathogenesis. Kisspeptin (KP) a G-protein coupled receptor neuropeptide, has been suggested as a link between energy balance and reproduction. Some studies have shown that the attenuation of KP signaling decreases metabolism and energy expenditure. KP mRNAs and receptors are detected in the hippocampus and cause the promotion of excitatory synaptic responses through modulation of postsynaptic signaling. The purpose of this study was to investigate the effect of KP on spatial learning and memory and its possible neuroprotective effect on Amyloid-Beta induced cognitive impairment using the Morris Water Maze (MWM) task in rats. The reference and reversal spatial learning and memory have been measured in this study. Rats were injected bilaterally by Aβ1-42 (2 μg/μL) or saline as a vehicle into the hippocampal CA1 area. One week later, KP-13 (1.5 or 2 µg/µL) was injected i.c.v before or after each training session for 3 days and memory was tested 24 h later. The results showed KP-13 by itself could significantly enhance spatial memory consolidation and retrieval, and Aβ induced reversal and reference memory impairment was significantly ameliorated by KP-13. In Conclusion, it seems that KP-13 as a neuropeptide has to enhance spatial memory properties and could be a possible neuroprotective peptide on amyloid-beta induced pathology.

## Introduction

Alzheimer’s disease (AD) as a neuro-degenerative disease causes a common form of dementia and causes progressive degradation in neuronal synapses (1). AD results in the continuing loss of neurons, synaptic dysfunction, disrupting communication within neural circuits in the medial temporal lobe, specifically in the entorhinal cortex and hippocampus that are important for memory and other cognitive functions (2, 3). 

Studies have shown hippocampal CA1 pyramidal neurons which have the main role in processing memory, are severely damaged during AD (4). Also, spatial memory impairment and attenuating of long-term potentiation (LTP) has been detected in the CA1 and dentate gyrus in a transgenic mouse model of AD (5). In recent years, the relation between AD and metabolic disorders has been studied by many authors. Some studies show brain glucose metabolism impairments, obesity and diabetes may lead to cognitive decline and AD pathogenesis. Furthermore, pathological changes in metabolism signaling pathways and energy homeostasis result in a disruption in neuronal survival, modulation of neurotransmitter release, neuronal outgrowth, synaptic plasticity, gene expression and memory processes (6, 7). Besides, late events in AD include Aβ plaque formation, impaired glucose and insulin tolerance and memory loss (4). 

Kisspeptin (KP), formerly known as Metastin, a G Protein Coupled Receptor (GPCR) neuropeptide, is essential for reproduction, fertility, and puberty. Kisspeptin is a vital component for the neuroendocrine regulation of GNRH secretion. In vertebrate, four paralogous Kiss-R genes (namely, KissR1, KissR2, KissR3, and KissR4) have been described (8), but the only verified physiological ligands of Kiss1R are Kisspeptin, which is a group of peptides encoded by Kiss1 gene (9, 10). All Kisspeptin isoforms have been resulted from the proteolysis of the pre-prokisspeptin as a precursor. Proteolysis of this precursor provides a 54-amino acid peptide, kisspeptin-54 (KP-54), kisspeptin-10 (KP-10), kisspeptin-13 (KP-13), and kisspeptin-14 (KP-14). In humans, Kiss1R mRNA expression has been detected in several types of tissues including pituitary, spinal cord, pancreas, brain, stomach, small intestine, thymus, spleen, lung, testis, kidney, and fetal liver (9). In the brain, expression of Kiss1R mRNA has been found in many areas, including the basal ganglia, amygdala, substantia nigra, hippocampus, spinal cord and the hypothalamus (10).

Several studies have explained kisspeptin signaling as a link between energy balance and reproduction (11). Also, Kisspeptin containing neurons are found in the arcuate nucleus of the hypothalamus, the part of the brain where reproductive and metabolic cross-talk happens (12-14). Some studies have shown that attenuation of kisspeptin signaling decreases the metabolism and energy expenditure with an unknown mechanism (15). Kiss1 expression has been reported to be inhibited during negative energy balance and metabolic stress (16). In addition, Kiss-1 mRNA expression and Kiss1-R are detected in high density in the granule layer of the dentate gyrus (DG) and in the pyramidal cells of hippocampal CA1 and CA3 area (17) and causing promotion of excitatory synaptic responses through modulation of postsynaptic signaling (18). Therefore, it has been suggested that kisspeptin may play a role in cognition (19). Recent data revealed that kisspeptin has an essential role in the regulation of social behaviors such as mood, fear, aggregation, and anxiety, relevant to reproduction (20). 

There are few studies about KP-13 on learning and memory. Telegdy and Adamik reported improvement of memory consolidation by KP-13 on passive avoidance learning memory (21). Furthermore, Jiang and colleagues have shown improvement of memory in novel object recognition (NOR) and object location recognition (OLR) tasks by i.c.v and intrahippocampal injection of KP-13 in mice (22). However, the effects of KP-13 on spatial learning and memory has not been done so far. 

Jiang *et al.*, reported that KP-13 could prevent Aβ-induced memory impairment in mice in NOR and LOR tasks (22). Moreover, Pourmir *et al.*, have shown that KP-13 ameliorated streptozotocin (STZ) induced learning and memory impairment in rats (23). 

Since Kiss-1 mRNA expression and Kiss1-R are located in high density in different parts of the hippocampus, the main focus of this study was to investigate the effects of KP-13 on spatial learning and memory using Morris Water Maze (MWM) spatial navigation task (24), which is a hippocampal-dependent task. We also assessed whether KP-13 treatment prevents cognitive impairment-induced by β-amyloid in rats.

## Experimental

*Animals*

In this study male albino Wistar rats, weighed between 180 to 200 g, were used. They were maintained four per cage in a constant-temperature room (24 ± 1 °C), under 12-hours light/dark cycle (lights on at 07:00 h). All animals had free access to food and water. The experimental procedures have been done in accordance with the National Institutes of Health Guidelines (NIH) for the care and use of laboratory animals, and all efforts were made to minimize animal pain and decrease the number of animals used in the experiments. The experimental procedures were approved by the ethics committee of school of the medicine of Shahid Beheshti University of Medical Science under permit number 308-23690.

*Drug Administration*

Human β-Amyloid 1-42 (Abcam, USA) was prepared as stock solutions in 0.1 M phosphate-buffered saline (PBS; pH 7.4) and aliquoted (20 µL per vial) and stored at −20 °C until use. The peptide was still perfectly soluble after defrosting the aliquots and before the injection. Aβ was incubated in sterile saline at 37 °C for 7 days to reduce peptide’s toxicity (25). For each injection 2 μLof incubated β-Amyloid (2 μg/μL) was used. 

The animals were anesthetized with intraperitoneal ketamine (100 mg/kg) and xylazine (10 mg/kg). They were injected bilaterally under the stereotaxic surgery by Aβ or its vehicle (Saline) into the CA1 area of the hippocampus (AP: -3.6, L: ±2.4 and DV: -2.8), according to the Atlas of Paxinos and Watson (26).

Injections were made over 2 min using a 5 µL Hamilton syringe fitted with a 30-gauge blunt-tipped needle, and the needle remained in place for an additional 1 min before it was slowly retracted. After CA1 injection, animals were implanted with a cannula (8 mm, 23 gauge) located on the right ventricle (AP: −0.5 L: 1.6 DV: −4.2). The cannula was fixed to the skull with a screw and dental cement. Rats were injected (i.c.v) 1 µL of different doses of KP-13 (Bachem Basel, Switzerland) or saline, 30 min before starting each training sessions, or immediately after the training. Three doses of KP-13, 1, 1.5, 2 μg/µL were used, and each group received only one of them (21, 22). A 30-gauge needle attached to a 5 μL Hamilton syringe by a polyethylene tube was used for the injections.

*Morris water maze*

*Apparatus*

A pool with 155 cm in diameter and 60 cm high located in a dark room was used as a water maze. It was filled with water (21 ± 1 °C) until 10 cm from the edge of the tank. The transparent Plexiglas platform (10 cm diameter) 1.5 cm was located below the water surface in the tank, in center of one of the four quadrants (target quadrant, Q1). The platform was the only way out of the water. There were some fixed extra maze (spatial) cues on the walls surrounding the pool. These cues were visible to the animals, during the whole experiment to allow them to find the hidden platform. The CCD camera (Panasonic Inc., Japan) was hanging from the ceiling above the MWM apparatus to record animal’s movements, and the Ethovision software (version XT7, Netherland) was used for measuring locomotion tracking.

*Habituation*

Twenty-four hours before starting the first training, rats were given a 60 s swim in the tank without a platform for adaptation to the environment.

*Procedure*

*Reference spatial learning and memory*

Seven days after the surgery, training of the rats in the MWM was started. During three consecutive training days, the platform was submerged in the north quadrant (target quadrant). The platform location was fixed throughout training, but animals started swimming from different locations chosen from the west, east, southwest, and southeast randomly (while facing the tank wall). Each training session consisted of four trials with 10 min intervals in between. Animals had 60 s for swimming to reach the platform. The rats were directed to the platform if they could not find it within 60 s and were allowed to rest on it for 30 s. The spatial probe test was done one day after the 3rd session, and rats were allowed to swim for 60 s in the pool without the platform. Mean Escape latencies, percentage time spent in target quadrant were recorded with a video tracking system for subsequent analysis (27, 28).

*Reversal spatial learning and memory*

For the reversal learning and memory test, in the 5^th^ day, the day after the spatial probe test, the platform was placed in the opposite quadrant (southern quadrant). Rats swam to find the new platform location during two blocks. Each block was included of four trials with 10 min inter-trial interval and there was a 30 min break between the blocks. The Platform location was fixed at the same place throughout training and the rat was fallen down from different locations in each trial. In 6th day, the reversal probe test was done while the platform was removed and rats were allowed to swim for 60 s (29).

*Visio-motor Activity*

After the reversal probe trial, the platform was raised above the water surface and covered by aluminum foil and placed in a different zone. Rats swam and find the visible platform during 60 s in order to test their visio-motor ability.

*Hematoxylin-Eosin Staining*

To investigate the survival of hippocampal CA1 cells, histological studies were done using Hematoxylin-Eosin (H&E) staining. The rats were anesthetized with an i.p injection of ketamine (100 mg/kg) and xylazine (10 mg/kg) after the reverse probe test and perfused transcardially with 0.9% saline solution followed by 4% paraformaldehyde in phosphate buffered saline (PBS). The brain was removed and kept in the same fixator at 4 °C overnight. After 24 h two hemispheres were separated and the cerebellum and olfactory bulbs were cut. For better approaching to the hippocampus, the first 6mm of the frontal cerebral hemispheres was removed and the remaining part of the brain, including the hippocampus, was fixed in paraffin. The paraffin tissues blocks were sectioned (6 µm) serially and coronally using a microtome. The sections were placed in 37 °C overnight then kept in the 4 °C refrigerator until the HE staining. Before counting the cell, sections were stained with cresyl violet. The surviving cells had round-shaped nuclei, with an undamaged cytoplasmic membrane and no nuclear condensation or distortion. Live pyramidal cells distributed in the CA1 area of the hippocampus were detected using high magnification (100X).

*Statistics*

Different parameters of training days were analyzed by two-way analysis of variance (ANOVA) followed by post-hoc analysis (Bonferroni test) and the probe test parameters, were analyzed by one-way ANOVA followed by post hoc analysis (Tukey’s multiple comparisons test) using the GraphPad Prism® 5.0 software. Data were performed as mean ± SEM (Standard Error of Mean). A *p* < 0.05 was considered to be statistically significant.

## Results

*Effects of different doses of KP-13 on the reference and reversal spatial learning and memory*

**Figure 1:**
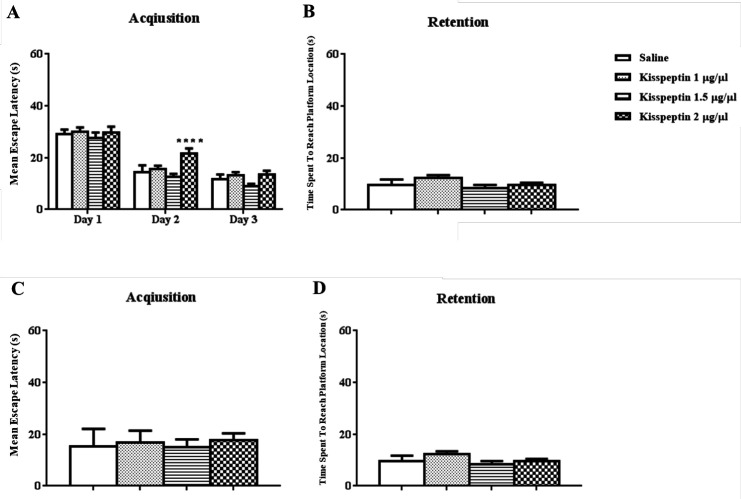
Effect of KP-13 on the reference and reversal spatial learning and memory. (A and B) Different doses of KP-13 had no significant effect on reference spatial learning and memory except there was a significant increase in the mean escape latency in the second day by 2 µg/µL of KP-13. (C and D) In the reversal spatial learning and memory test, there was no significant difference between the groups in the mean escape latency in the acquisition and in the retention test (C and D), ^**^p < 0.01 compared to the control group

The results of daily 1 µL i.c.v injection of KP-13 (1, 1.5, 2 μg/µL) before each training day and the probe trial test is illustrated in Figure 1. In training days two-way ANOVA of the mean escape latencies in acquisition showed significant day [F(2,72) = 144.7; *p *< 0.01] but no group effects [F(3,36) = 2.99; *p* = 0.04] except for the 2^nd^ day of training in 2 μg/µL of KP-13 *p* < 0.01 (Figure 1A). In addition, data analyses of memory retention test had no significant effect on the time spent to reach the platform location using one-way ANOVA [F(3,33) = 1.65; *p* = 0.19] (Figure 1B). In the reversal spatial learning and memory test, KP-13 did not significantly affect the mean escape latencies in the acquisition [F(3,33) = 0.74; *p* = 0.53], and in the retention test [F(3,33) = 0.41; *p* = 0.74], when analyzed by one-way ANOVA (Figures 1C and 1D). 

*Post-Training injection of KP-13 enhanced memory consolidation in the MWM task*

**Figure 2:**
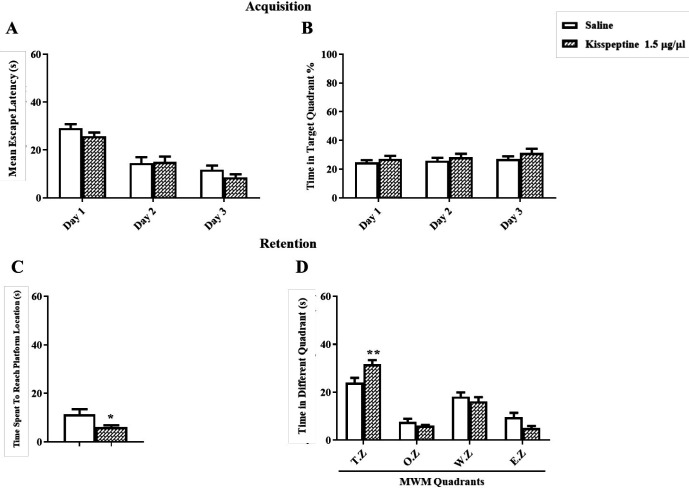
Effect of KP-13 on memory consolidation of the MWM test. (A and B) kisspeptin (1.5 µg/µL) treated group learned the task as the control group. (C and D) but the injection of KP-13 after training each day, significantly improved the memory consolidation comparing to the saline group as it is shown by decreasing the mean escape latency and increasing the time spent in the target zone. ^*^p < 0.05, ^**^p < 0.01 compared to the control group. T.Z: Target Zone; O.Z: Opposite Zone; W.Z: West Zone; E.Z: East Zone

For testing the effect of KP-13 on spatial memory consolidation, five minutes after training, KP-13 (1.5 µg/µL) was injected. In training days two-way ANOVA of the mean escape latencies showed significant day [F(2,36) = 67.36; *p *< 0.01] but no group effects [F(1,18) = 1.03; *p* = 0.32] (Figure 2A). In addition, the percentage of time spent in the target zone during training days using two way ANOVA [F(2,36) = 0.12; *p* = 0.88] was not affected (Figure 2B). But data analyses of memory retention test using *t*-test (t = 2.41, df = 13, *p* = 0.03) had a significant effect on the time spent to reach the platform location and the time spent in the target zone using two-way ANOVA [F(3,48) = 4.69; *p* = 0.005] in the probe test (Figures 2C and 2D). 

*Reference spatial memory was affected by CA1-Aβ pathogenesis*

**Figure 3:**
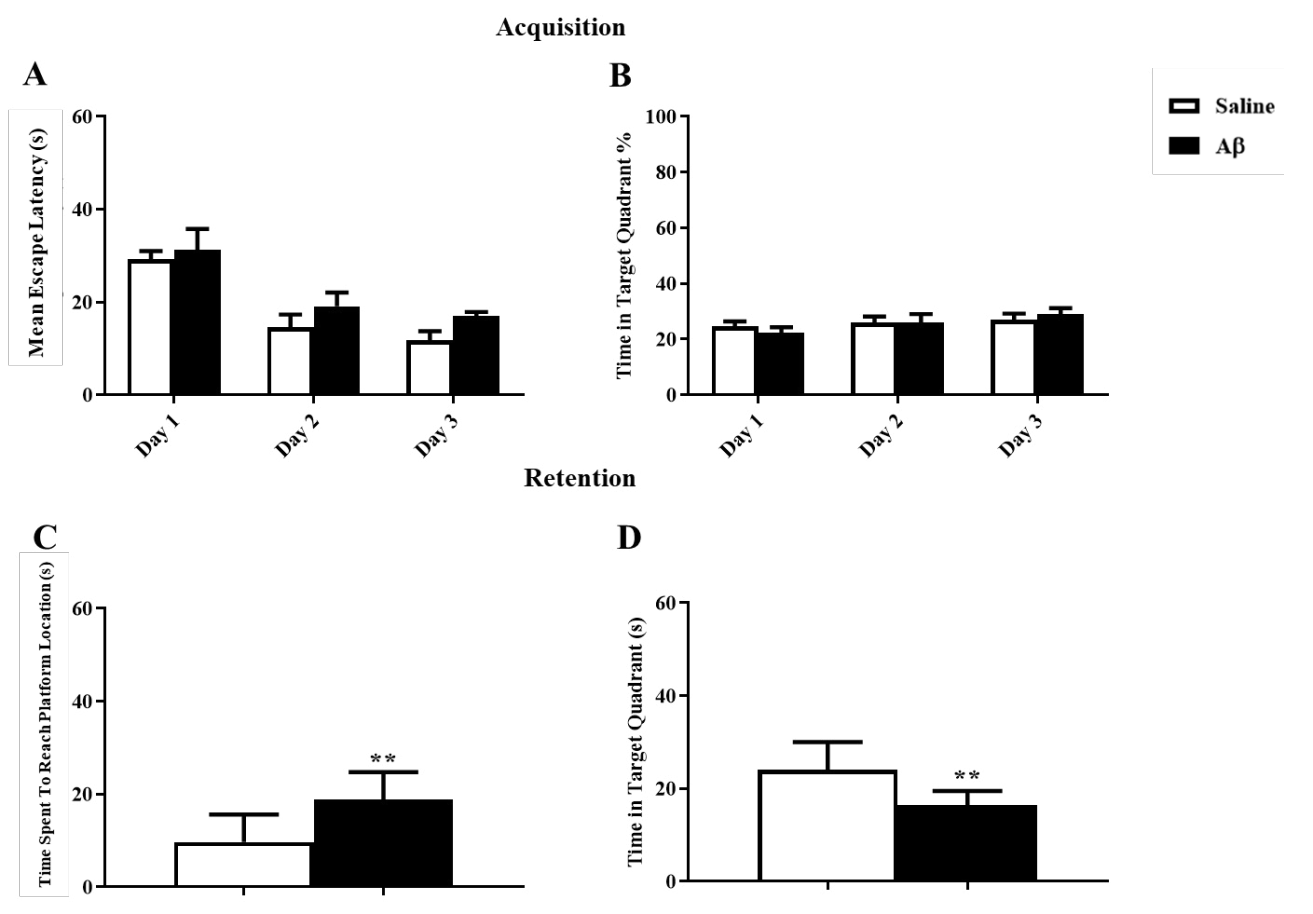
Effect of Amyloid Beta on reference learning and memory. (A and B) There was no significant effect on the reference learning test. (C and D) Aβ significantly damaged memory retention, ^**^p < 0.01 compared to the control group

For induction of AD model, Aβ was injected in the hippocampal CA1, and after 7 days the reference spatial learning was assessed in the MWM task. As it is shown in Figures 3A and 3B, the mean escape latency and the percentage of time spent in the target zone during training days using two way ANOVA [F(1,16) = 1.98; *p *= 0.17] and [F(1,16) = 0.0025; *p* = 0.96] were not affected in the Aβ treated group respectively. However, in the probe test, Aβ significantly impaired memory retention by increasing the time spent to reach the platform and decreasing the time spent in the target zone using unpaired *t*-test (t = 3.26, df = 16, *p* = 0.005) and (t = 3.4, df = 16, *p* = 0.004) respectively (Figures 3C and 3D).

*Aβ-treated rats showed a deficit in the reversal reference spatial learning and memory*

**Figure 4:**
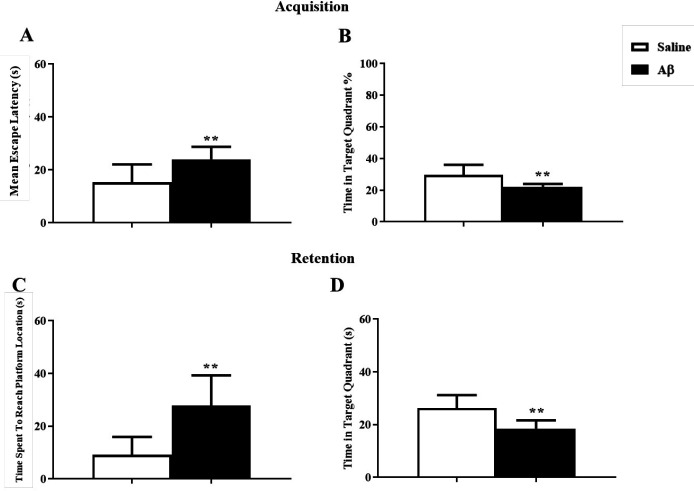
The effect of Aβ on reversal reference learning and memory. (A and B) Aβ had a significant deteriorating effect on the reversal reference learning test. (C and D) Aβ impaired the reversal reference memory retention ^**^p < 0.01 compared to the control group

Analysis of data showed that reversal reference learning and memory had been disrupted by Aβ treatment. In the training test, Aβ significantly impaired acquisition, changing the mean escape latency and the percentage of time spent in the target zone using unpaired *t*-test (t = 3.2, df = 15, *p* = 0.005) and (t = 3.33, df = 15, *p* = 0.004) respectively (Figures 4A and 4B). In the probe test, in the Aβ treated group the time spent to reach the platform and the time spent in the target zone were significantly changed using unpaired *t*-test (t = 4.038, df = 15, *p *< 0.01) and (t = 3.99, df = 15,* p *< 0.01) respectively (Figures 4C and 4D).

*KP-13 decreased the impairments due to Amyloid Pathology in the reference probe test*

**Figure 5:**
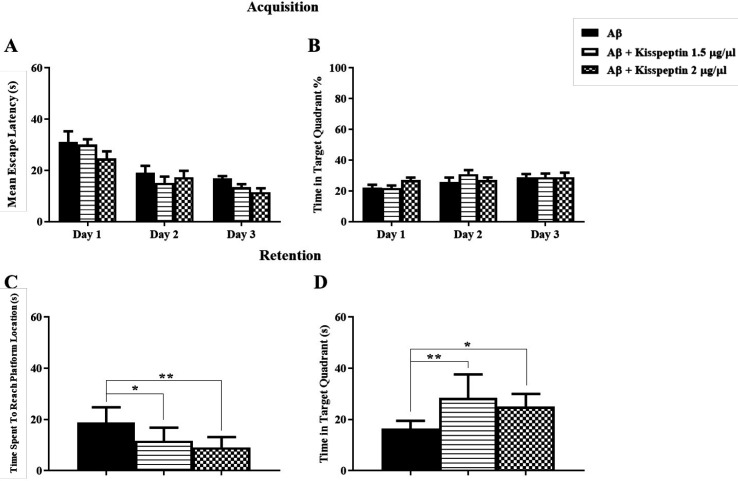
Effect of different doses of KP-13 on reference spatial learning and memory of Aβ treated rats. (A and B) There was no significant effect on the mean escape latency and the percentage of time spent in the target zone by KP-13 in the training days. (C) In the probe day there was a significant difference between the groups in the time spent to reach the platform and (D) on the time spent in the target zone, ^*^p < 0.05, ^**^p < 0.01 compared to the Aβ group

To investigate the effects of KP-13 on Aβ treated rats, two doses of KP-13 (1.5 and 2 µg/µL) were used. Seven days after the Aβ injection, rats received daily i.c.v injection of KP-13 before each training day. As shown in Figure 5A using two-way ANOVA the mean escape latencies in the acquisition phase showed significant day [F(2,52) = 35.23; *p* < 0.0001] but no group effects [F(2,26) = 2.12, *p* = 0.13] in rats received KP-13, 7 days after the Aβ injection compared to the Aβ group. Also the percentage of time spent in the target zone in training days, a two-way ANOVA indicated significant day [F(2,54) = 4.68; *p *= 0.01] but no group effect [F(2,27) = 0.65; *p *= 0.52] (Figure 5B). However, in the probe test, data analyzes showed KP-13 in both doses significantly improved the Aβ deficit induced in the reference memory. One way ANOVA test [F(2,25) = 8.87; *p* = 0.0012] indicated KP-13 significantly decreased mean latency of the first pass to the platform location which has been increased by Aβ (Figure 5C). In addition, KP-13 could significantly increase the time spent in the target quadrant in the Aβ treated rats, using one way ANOVA [F(2,25) = 8.87;* p *= 0.0016] (Figure 5D). Consequently, KP-13 in both doses alleviated Aβ pathogenesis in the spatial reference memory. 

*KP-13 diminished the reversal spatial learning and memory deficit caused by CA1 amylopathy*

**Figure 6:**
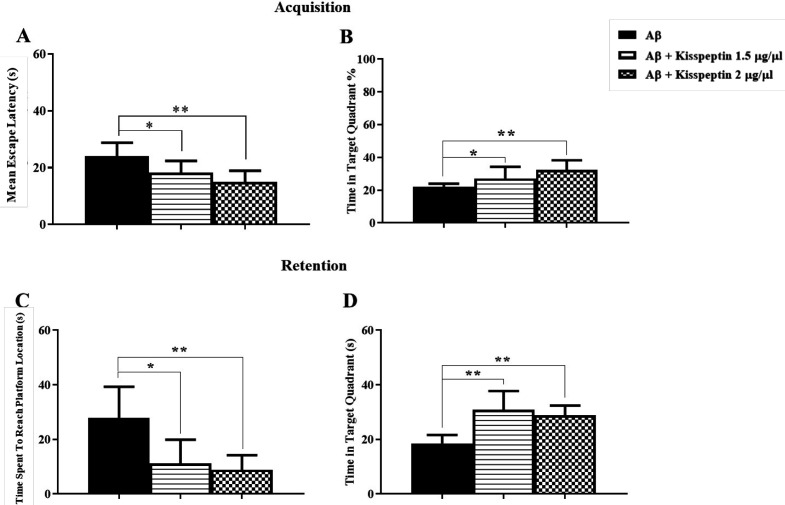
Effect of KP-13 on the reversal spatial learning and memory disruption due to Aβ. (A and B) There was a significant decrease in the mean escape latency and an increase in the percentage of time spent in target zone using 1.5, 2 µg/µL KP-13. (C and D) In the reversal probe test, the time spent to reach the platform location significantly decreased and the time spent in the target zone increased significantly by both doses. ^*^p < 0.05, ^**^p < 0.01compared to the Aβ group

As shown in Figures 6A and 6B in the training day the mean escape latency to platform significantly decreased by both doses of KP-13 compared to the Aβ group using one way ANOVA test [F(2,24) = 10.47; *p *= 0.0005] (Figure 6A). Also, one-way ANOVA [F = 8.19; *p* = 0.0019] indicated daily microinjection of both doses of KP-13 could significantly diminish the Aβ effect on the percentage of time spent in the target zone (Figure 6B). Twenty-four hours after reverse training, the platform was removed and animals were permitted to swim for 60 s. As shown in Figure 6C, in the reverse probe day, one-way ANOVA (n = 27; *p* = 0.0007) indicated the time spent to reach the platform in the Aβ treated rats was significantly much longer than the groups treated by KP-13. Ultimately the time spent in the target zone reduced by KP-13 in both doses compared to the Aβ group, using one way ANOVA [F(2,24) = 17.41; *p *< 0.0001] (Figure 6D).

*Visible platform test and swimming speed analysis*

**Figure 7:**
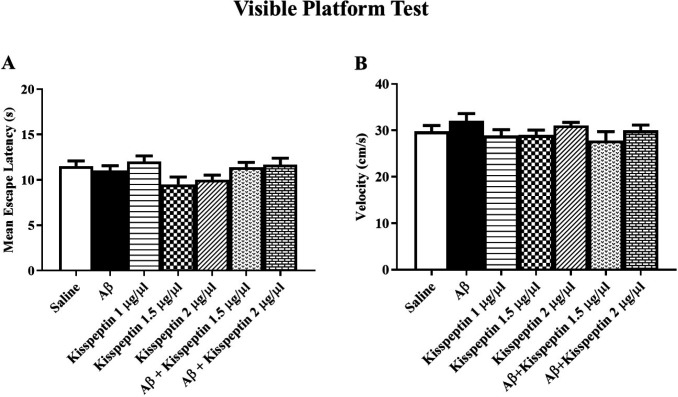
(A) There was no significant difference in the escape latency between groups in the visible text. (B) There was also no significant difference in the swimming speed between groups (n = 9 each group)

In the visible test which was taken after the probe test, all groups could find the platform (Figure 7A) indicating no visual damage in the animals [F(6,58) = 2.17: *p* = 0.058]. The swimming speed did not show any significant variation in the probe test (Figure 7B) indicating no motor disturbance in the experimental animals [F(6,57) = 1.25; *p* = 0.29]. 

*KP-13 significantly decreased the neuronal loss in the hippocampus of Aβ treated rats*

**Figure 8:**
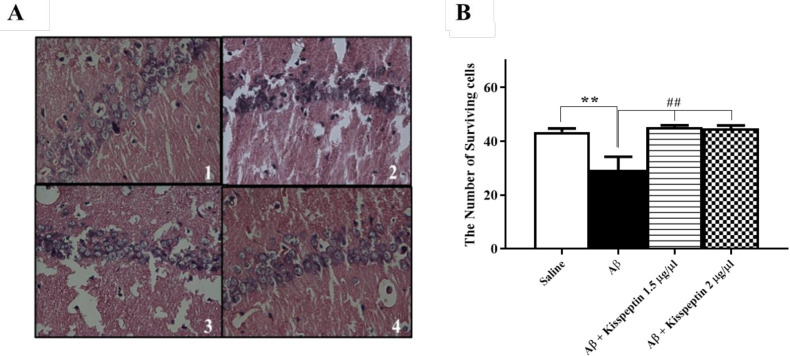
(A) Representative photomicrographs of hematoxylin-eosin–stained hippocampal CA1 pyramidal neurons 1: Saline, 2: Aβ, 3: Aβ+Kisspeptin 1.5 µg/µL, 4: Aβ+Kisspeptin 2 µg/µL. (B) Effect of KP-13on hippocampal neuronal loss in the Aβ treated rats. KP-13 significantly increased the survival of CA1 cells in the Aβ treated rats. Photographs were taken at 40X magnification. ^**^p < 0.01 compared to the saline group, ^##^*p* < 0.01 compared to the Aβ group

H&E staining was used to identify neuronal alterations in the hippocampus. The results showed an increasing number of dead cells in the CA1 area of the Aβ group compared to the control group (Figure 8A). Data analyses of cell counting showed that KP-13 in all doses could significantly alleviate the neural loss in the Aβ treated compared to the control rats [F(3,11) = 11.24; *p* = 0.0011] (Figure 8B).

## Discussion

In this study, the possible neuroprotective role of KP-13 in spatial learning and memory impairment due to injection of intra-hippocampal amyloid-beta (1-42) in rats was investigated. Our results showed that different doses of KP-13 had no effect on spatial learning but it improved the memory consolidation of MWM. Moreover, KP-13 alleviated the reference memory impairment in the Aβ treated rats. In addition, it prevented the reversal spatial learning and memory deficit due to Aβ treatment. Furthermore, H&E staining indicated that KP-13 could significantly prevent the neural loss in the Aβ treated compared to the control rats.

In our experiments, KP-13 had no effect on the acquisition of MWM which is in agreement with Delmas *et al.*, findings (30). Our results exhibited that KP-13 has a significant effect on consolidation and retention of MWM. It has to be mentioned that there is no report indicating spatial memory consolidation and retention enhancement by KP-13 in MWM so far. However, there is one report by Telegdy and Adamik, indicating KP-13 had an impressive effect on the consolidation of passive avoidance learning and memory (21). Also, Jiang *et al.* showed that KP-13 amplify object memory formation and extends memory retention through stimulation of GPR54 in the hippocampus (22).

Kisspeptin containing neurons and receptors (KISS1R) are identified throughout the brain including the hippocampus, proposing that KP is important in organizing the activity of multiple neuronal circuits (10). The anatomical and lesion studies revealed that hippocampus has an essential role in managing spatial short and long term memory (31). One study showed that activation of GPR54 by KP-13 reversibly increases excitatory synaptic transmission in hippocampal dentate granule cells (32). In addition, it has been reported that treatment with KP increases the firing rate of hippocampal cells (18, 32 and 33). It has been suggested that treatment of hippocampal cell culture with KP increase the expression of mRNA of BDNF (18), which probably leads to enhancing learning and memory formation (33). Since, KP-13 has interactions with many neuropeptides which are involved in learning and memory processes including aminergic, GABA-ergic, cholinergic and GnRH receptor systems (21, 23), the functionality of KP-13 might be through these receptor systems which should be further investigated.

Studies on post mortal human brain and *in-vivo* fMRI have revealed that the CA1 and the subiculum are the most damaged areas in AD (34, 35). In our experiment, Aβ injection in CA1 could damage the reference and reversal spatial memory, which is in agreement with several reports indicating the role of CA1 in spatial memory impairment due to Aβ neurotoxicity (29, 36-38). Moreover, there are many findings of the potential relationship between AD and metabolic disorders like obesity and diabetes (39, 40). These findings indicate that brain glucose metabolism deficiencies can lead to AD pathogenesis (40). In fact, it has been suggested that AD is a “type 3 diabetes” metabolic disorder and, some antidiabetic agents may have a pro-cognitive effect on AD (41, 42). One report using high-resolution PET and MRI in 2018 suggested that glucose metabolism was significantly decreased in the CA1 region of the hippocampus in AD (43). As previously mentioned, the decrease of KP signaling results in brain metabolism decline and energy expenditure (11, 15).

In the current study, we found KP-13 could alleviate the impairments in reference to spatial memory due to Aβ pathology. Moreover, Aβ-treated rats showed a deficit in the reversal reference spatial learning and memory, which were diminished by KP-13. Similar to our results but in two other cognitive tasks, NOR and LOR, Jiang *et al.*, in 2015 reported KP-13 improves memory deficiency made by Aβ1–42. Also, they showed that this effect is through KP-13 receptor (GPR-54) (22). In another study i.c.v injection of KP-13 facilitated spatial learning and memory in streptozotocin-induced AD (23). It has to be mentioned that Aβ (1-42) and streptozotocin (STZ) mimic many pathological aspects of human AD and both models are frequently used. However, Aβ is mostly used to induce early-onset AD (44), and STZ is used to induce late-onset AD (44, 45). Singh and Kumar comparing the potency of STZ and Aβ models have revealed that Aβ (1-42) is more specific and dependable for AD cognitive dysfunction and histopathological alterations in experimental animals. However, it has been suggested that Aβ is significantly more potent in memory disruption, production of oxidative damage and neuroinflammation than STZ, suggesting its reliability in AD studies (46). In our study, Aβ was injected one week before starting experiments producing an early-onset AD.

Milton and coworkers, working on human neural cells, showed that KP ameliorates Aβ toxicity by direct binding to Aβ, not through its receptor (GPR-54) (47). Whether the effect of KP-13 on learning and memory and also its neuroprotective effect against Aβ toxicity is through its receptor or its direct binding to Aβ, needs to be further investigated.

In conclusion, the results of this study illustrate the spatial memory enhancing effect of KP-13 and also, the neuroprotective effect of this peptide on Aβ cognitive dysfunction.
